# Anatomical acclimation of mature leaves to increased irradiance in sycamore maple (*Acer pseudoplatanus* L.)

**DOI:** 10.1007/s11120-022-00953-4

**Published:** 2022-09-03

**Authors:** Tomasz P. Wyka, Piotr Robakowski, Roma Żytkowiak, Jacek Oleksyn

**Affiliations:** 1 General Botany Laboratory, Institute of Experimental Biology, Faculty of Biology, Adam Mickiewicz University, ul. Uniwersytetu Poznańskiego 6, 61-614 Poznań, Poland; 2 Poznań University of Life Sciences, ul. Wojska Polskiego 71a, 60-625 Poznań, Poland; 3 Institute of Dendrology, Polish Academy of Sciences, ul. Parkowa 5, 62-035 Kórnik, Poland

**Keywords:** Forest gaps, Palisade mesophyll, Photo-oxidative stress, Shade adapted trees, Spongy mesophyll, Tree regeneration

## Abstract

Trees regenerating in the understory respond to increased availability of light caused by gap formation by undergoing a range of morphological and physiological adjustments. These adjustments include the production of thick, sun-type leaves containing thicker mesophyll and longer palisade cells than in shade-type leaves. We asked whether in the shade-regenerating tree *Acer pseudoplatanus*, the increase in leaf thickness and expansion of leaf tissues are possible also in leaves that are already fully formed, a response reported so far only for a handful of species. We acclimated potted seedlings to eight levels (from 1 to 100%) of solar irradiance and, in late summer, transferred a subset of them to full sunlight. Within 30 days, the pre-shaded leaves increased leaf mass per area and became thicker mostly due to the elongation of palisade cells, except for the most shaded individuals which suffered irreversible photo-oxidative damage. This anatomical acclimation was accompanied by a transient decline in photosynthetic efficiency of PSII (F_v_/F_M_), the magnitude of which was related to the degree of pre-shading. The F_v_/F_M_ recovered substantially within the re-acclimation period. However, leaves of transferred plants were shed earlier in the fall, indicating that the acclimation was not fully effective. These results show that *A. pseudoplatanus* is one of the few known species in which mature leaves may re-acclimate anatomically to increased irradiance. This may be an important mechanism enhancing utilization of gaps created during the growing season.

**Supplementary Information:**

The online version contains supplementary material available at 10.1007/s11120-022-00953-4.

## Introduction

Survival and growth of trees regenerating in the understory is made possible by adjustments of plant architecture and leaf anatomy, and physiological regulation, together increasing the efficiency of light harvesting and improving the whole plant’s energy budget (Givnish 1988; Valladares and Niinemets 2008). Especially important is the acclimatory modification of leaf structure and physiology, allowing optimization of photosynthetic capacity with respect to light harvesting (Oguchi et al. 2005). Anatomical acclimation to low irradiance manifests itself in the production of thin leaves containing single-layered palisade mesophyll, with short cells and a small number of chloroplasts. In contrast, leaves produced under high light often contain several layers of strongly elongated palisade cells, capable of accommodating large numbers of chloroplasts needed for absorption and utilization of the incoming energy (Terashima et al. 2005). While shade leaves tend to be richer in chlorophyll and contain a similar amount of nitrogen on a mass basis as sun leaves, the sun leaves, because of their greater thickness, contain more nitrogen on an area basis and have a reduced chlorophyll/nitrogen ratio, reflecting the shifting balance between factors limiting photosynthesis (Poorter et al. 2019). Other structural modifications in shade may include lower vein and stomatal density (Scoffoni et al. 2015; Poorter et al. 2019), reduced development of features associated with phloem loading (Amiard et al. 2005), and increased lamina size as well as reduced lamina lobation or dentation (Nicotra et al. 2011), relative to sun leaves.

Leaves are determinate organs, with an early stage of area expansion and tissue formation lasting usually up to 6 weeks in temperate trees (Niinemets et al. 2004; Ding et al. 2014) followed by a developmental plateau (Pantin et al. 2012). According to this accepted view, formation of the final leaf shape, thickness, and tissue structure takes place during its early stage of growth, and structural acclimation to light must take place during this early time window. Yet, the light environment in the temperate forest canopy is dynamic, with, e.g., gradual canopy defoliation increasing the penetration of light late in the season. Occasional formation of canopy gaps, whether natural or man-made, causes wholesale increases of irradiance in the understory. In fact, gap formation is required by many shade-tolerant trees for growth into the canopy layer. Such sudden increases in irradiance may cause photo-oxidative stress in unacclimated leaves (Oguchi et al. 2006), setting off complex defensive and acclimative responses (Tognetti et al. 1998; Campa et al. 2017; Oguchi et al. 2017) or causing premature leaf death. Developmental plasticity allows new leaves of understory plants produced after an increase in irradiance to match the new conditions physiologically and structurally. On the other hand, in pre-existing leaves, the extent of physiological, particularly photosynthetic, re-acclimation varies among species (Yamashita et al. 2000; Calzavara et al. 2017; Oguchi et al. 2017; Martinez and Friedley 2018) and anatomical re-acclimation is considered rare (Oguchi et al. 2018).

Only a handful of studies appear to have actually tested the ability of mature, fully expanded leaves to adjust anatomically to increased irradiance. Although such leaves may respond to increased irradiance by increasing leaf mass per area (LMA), this usually occurs in the absence of any significant increase in thickness (Naidu and DeLucia 1997; Yamashita et al. 2000; Yamashita et al. 2002; Niinemets et al. 2003; Oguchi et al. 2006 but see Frak et al. 2001). Such increases in LMA could be explained by accumulation of carbohydrates and various other protoplast components, as well as cell wall material, leading to increased leaf tissue density. In contrast, the majority of studies that examined the anatomical traits that typically contribute to sun vs. shade leaf anatomy (such as lamina thickness, mesophyll or epidermal thickness or palisade cell length), showed a lack of adjustment of these traits. Examples include seedlings of shade-tolerant trees: *Acer saccharum* and *Quercus rubra* (Naidu and DeLucia 1998), *Fagus sylvatica* (Tognettti et al. 1998), *Abies grandis*, *A. lasiocarpa* and *Picea engelmannii* (Youngblood and Fergusson 2003), *Betula ermanii* and *F. crenata* (Oguchi et al. 2005), *Q. petraea* and *Q. cerris* (Rodriguez Calcerrada et al. 2008), six other species of trees and two lianas (Oguchi et al. 2006) as well as herbs *Alocasia macrorrhiza* (Sims and Pearcy 1992), *Chenopodium album* (Oguchi et al. 2003), and *Cucurbita pepo* (Amiard et al. 2005). Notable exceptions, however, have been reported. Thickness of fully expanded leaves increased in response to increased irradiance in *Glycine max* (Bunce et al. 1977), *Bischofia javanica, Trema orientalis* and *Schima mertensiana* (Yamashita et al. 2002), *Pisum sativum* and *Verbascum phoeniceum* (Amiard et al. 2005), and *Acer rufinerve* (Oguchi et al. 2005). In *G. max, P. sativum, V. phoeniceum*, and *A. rufinerve*, the increase in lamina thickness was explainable by an increase in mesophyll thickness, which in *A. rufinerve* was accompanied by an increased area of cell walls contacting intercellular spaces. Another type of anatomical adjustment, found in *B. ermanii* and *A. rufinerve*, as well as in *C. album* involved the expansion of surface area of chloroplasts leading to their increased contact with cell walls and resulting in enhanced mesophyll conductance to CO_2_ (Oguchi et al. 2003, 2005). Moreover, in a single case documented till date, palisade cells in shade-acclimated leaves of *Hedera helix* exposed to an increased irradiance underwent divisions leading to the formation of an additional palisade layer (Watson 1942; Bauer and Thöni 1988). Results of the above studies indicate that the capacity of fully developed leaves for anatomical acclimation to increased light is species-specific and possibly rather rare. On the other hand, it appears that only a small number of species have been investigated using an experimental design that analyzed anatomical changes in mature leaves after a shift in irradiance. Moreover, it may be expected that the extent of re-acclimation should depend on the original growth irradiance, as a high magnitude increase in light intensity may trigger photo-oxidative stress leading to cellular damage and lack of response. Applying multiple levels of growth irradiance may thus be useful in experiments tracking anatomical responses of mature leaves.

In this study, we examine the ability for anatomical re-acclimation of leaves of sycamore maple (*Acer pseudoplatanus* L.), a shade-tolerant winter deciduous tree with a broad distribution in Europe. Typical of shade-tolerant species, *A*. *pseudoplatanus* shows high survival and slow growth at low light intensities, a low photosynthetic rate at high irradiance, and a low light compensation point (Hättenschwiler and Körner 2000; Kazda et al. 2004). Seeds germinate usually before forest canopy closure in the spring but seedlings establish and survive under deep canopy shade. Higher irradiance is, however, required if they are to advance to the canopy layer (Helliwell and Harrison 1979). Remarkably, on fertile forest soil, seedlings can survive more than 15 years under deep shade (Hein et al. 2009). Sycamore maple is relatively tolerant to late spring frosts which explains the success of its establishment after the formation of large canopy gaps (Piovesan et al. 2005). We grew potted seedlings of sycamore maple under eight irradiance levels, ranging from deep shade to full sunlight. We then exposed plants to full solar irradiance and examined their response after 30 days to (1) test the hypothesis that mature leaves formed under the various growth irradiances are capable of anatomical acclimation to increased irradiance. We additionally (2) analyzed other responses indicative of acclimation (leaf nitrogen concentrations and photochemical efficiency of photosystem II) and (3) monitored leaf persistence on the plants for the remainder of the growing season to evaluate the extent of permanent damage caused by photo-oxidative stress.

This is a corrected version of a retracted paper (Wyka et al. 2022) that originally also analyzed the effect of increased irradiance on photosynthetic pigments. However, following the original publication, we discovered major discrepancies between our pigment levels and ratios, and those reported in literature for this and other species. Since the problem was attributable to an analytical error on our part, pigment data and the related discussion have been removed from the current version of the paper.

## Materials and methods

### Plant cultivation

In late April 2009, 80 two-year-old cold-stored, dormant bare-rooted seedlings of *Acer pseudoplatanus* L. were obtained from Poznań University of Life Sciences experimental nursery in Zielonka. Plants were transported to an experimental garden at Polish Academy of Sciences Institute of Dendrology in Kórnik (Poland, 55° 15′ N 17° 6′ E). Plants were immediately potted into 5 L pots filled with commercial peat-based compost with 10% (v/v) addition of topsoil from a mature *A.pseudoplatanus* stand and 3 kg m^− 3^ of slow-release fertilizer (Osmocote 15-10-12), and temporarily placed in a shaded spot. Eight levels of growth irradiance were created by erecting outdoor shade house frames and covering them with polyethylene shading cloth of different densities according to the manufacturer’s specifications or by combining cloth layers. Relative irradiance in each shade house was determined by taking simultaneous measurements of photosynthetic photon flux density (PPFD, covering wavelengths between 400 and 700 nm) inside the house and under open sky using two sensors (Apogee Instruments, Utah, USA) that gave nearly identical readings (*N* = 5 readings per house, Supplementary Information Table S1). Measurements were conducted on a sunny day with average (± SD) outside PPFD = 1570 ± 254 µmol m^−2^ s^−1^ (n = 35). Average relative irradiances for the different houses were 1.1%, 3.4%, 4.6%, 12.7%, 21.5%, 29.4%, and 49.6% of full solar irradiance (hereafter rounded to full numbers). On May 4, when buds were beginning to break, plants were randomly distributed among the shade houses (*N* = 10 plants for each). An additional set of 10 plants were left unshaded (100% irradiance). Average plant height at that time was 37.6 ± 12.9 cm (range 12.5–74 cm; Supplementary Information Table S2) and there was no significant difference in mean height among irradiance levels (Anova F_7;79_ = 1.09, *P* = 0.380). During the experiment, plants were watered regularly to field capacity. They were rearranged haphazardly within each shade house at 3–4-week intervals and kept apart to minimize mutual shading. In early August, when all terminal buds had formed, a leaf sample was collected from each plant (hereafter the PRE treatment) and half of the plants from each shade level were randomly selected and transferred to full irradiance (hereafter, the transferred group, TRANS), with the rest remaining under original growth irradiance (the control group, CONT), and maintained for the rest of the season. Daily minimal and maximal air temperatures and direct sunshine hours for the location were obtained from a local weather station (Supplementary Information Fig. S1).

### Chlorophyll fluorescence

Chlorophyll *a* fluorescence was measured in the morning on August 6, i.e., immediately prior to transfer of plants to full irradiance (day 0), then on the following day (day 1) and finally on September 4 (day 29 after transfer) using a Plant Efficiency Analyzer (PEA, Hansatech, Norfolk, UK). Measurements were conducted using the same permanently marked leaves (one per plant). In the evening before measurements, darkening clips were placed on the leaves. The following morning, the PEA fiberoptic was inserted into the clip and a 1 s pulse of saturating light (4000 µmol m^− 2^ s^− 1^) was delivered to the leaf surface. Maximal quantum yield (F_V_/F_M_) was calculated from the leaf fluorescence emission according to the formula F_V_/F_M_ = (F_M_-F_0_)/F_M_, where F_M_ is maximal fluorescence under saturating pulse and F_0_ is fluorescence in dark-adapted state (Maxwell and Johnson 2000).

### Leaf sampling

On August 5, i.e., one day before transfer to full irradiance*,* a leaf from one of the lower nodes was collected from each plant. Care was taken to select a leaf that was not shaded by other leaves of the same plant. A ca. 6 cm^2^ segment not containing midrib was first cut for determination of leaf mass per area. A 5 × 2 mm fragment was excised from the middle part of the lamina, excluding major veins, for anatomical analysis. Remaining leaf material, after removing major veins, was dried and used for determination of nitrogen and carbohydrates. Sampling was repeated on September 5 (30th day after transfer), where possible using leaves from equivalent positions on the stem.

### LMA and leaf anatomy

The exact area of the ca. 6 cm^2^ leaf segment was determined using a desktop scanner. The sample was dried in a forced circulation drier at 65 °C for 72 h and weighed. Leaf mass per area (LMA, g m^− 2^) was determined as ratio of lamina mass to lamina area after subtracting the content of non-structural carbohydrates from lamina mass. The 5 × 2 mm leaf fragments were fixed overnight at 4 °C in a solution consisting of 2% glutaraldehyde and 2% paraformaldehyde in 0.1 M phosphate buffer (pH 7.0). Samples were then dehydrated in a graded ethanol series, transferred to acetone and infiltrated with Spur’s resin in a stepwise manner using three overnight incubations in solutions of resin in acetone at graded concentrations followed by 48 h of incubation in 100% resin. Infiltration steps were conducted at 4 °C followed by polymerization in molds at 45 °C, 60 °C, and 75 °C for 48 h at each temperature. The embedded tissue was cut to 2-µm-thick sections using an ultramicrotome (Leica, Austria). Sections were stained with 0.1% toluidine blue in 1% borate buffer and photographed through a light microscope (Axioskop, Zeiss, Germany) using Powershot G5 digital camera (Canon, Japan). Anatomical measurements (thickness of palisade and spongy mesophyll and of each epidermis) were taken from digital images using LSM510 software (Zeiss, Germany). Two sections per leaf were analyzed and results were averaged.

### Carbohydrates and N

Leaf material was dried as above and ground to fine powder using a Culatti mill (IKE Labortechnik Staufen, Germany). The concentrations of total non-structural carbohydrates (TNC, the sum of reducing sugars, and starch) were determined by a modification of the method described by Haissig and Dickson (1979) and Hansen and Møller (1975). Sugars were extracted in methanol–chloroform–water, and tissue residuals were used for determination of starch content. Soluble sugars were determined colorimetrically with anthrone reagent at 625 nm within 30 min. Starch in the tissue residue was gelatinized and converted to glucose with amyloglucosidase. Glucose concentrations were measured using the peroxidase-glucose oxidase-o-dianisidine dihydrochloride reagent. The absorbance was measured at 450 nm after 30 minutes of incubation at 37 °C against glucose standards. For determination of nitrogen concentration, powdered tissue was subjected to analysis in an Elemental Combustion System CHNS-O 4010 (Costech Instruments, Italy/USA). Nitrogen concentration was expressed on a non-structural carbohydrate-free leaf mass basis (N_mass_) and a leaf area basis (N_area_).

### Leaf shedding

Prior to the beginning of autumnal leaf shedding, petioles of 10 leaves per plant were tied to the stem using dental floss to facilitate keeping track of individual leaves. Care was taken to include only leaves from lowest nodes, i.e., those formed before the transfer. Abscised leaves were censused at 3–4-day intervals until all selected leaves were detached from stems. The percentage of leaves retained on the plants in each treatment was calculated for a given day. Mean day-of-year of abscission was calculated for each growth irradiance in the TRANS and the CONT group.

### Statistics

Individual plants were considered independent replicates, with typically 10 samples representing the PRE treatment and 5 samples in each TRANS and CONT group. All plants survived the experiment, however, sporadic sample mishandling resulted in the final sample number being lower in some treatments and for some variables (Supplementary Information Table S3). Since plants from the lowest (1%) irradiance level lost all leaves upon transfer to full irradiance, and because the 100% irradiance plants were not subjected to transfer, our experimental set-up was unbalanced. For analysis of variance, we therefore excluded the 1% irradiance plants (but still show the data). Additionally, we randomly divided the 100% irradiance plants into two subgroups, assigning one as TRANS and the other as CONT, to produce a complete factorial design. This was, however, only possible for LMA and chemical traits, for which all plants were sampled. For anatomical analysis, only 5 plants were sampled from the 100% irradiance plants, we therefore excluded this group from analysis of variance and for anatomical traits conducted Anova on growth irradiances from 3 to 50% (but still show the complete data). We thus applied two-way Anovas with growth irradiance at six or five levels and three types of transfer treatment (PRE, CONT, and TRANS), followed by pre-planned contrasts between treatments within each growth irradiance. For growth irradiances not included in the Anova design (i.e., 1% irradiance for both anatomical and N concentrations, and 100% irradiance for anatomical traits), we calculated contrasts between PRE versus CONT plants. Significant contrasts between PRE and CONT plants indicated change in time, whereas contrasts between CONT and TRANS plants indicated the effect of an increased irradiance. Data were log transformed prior to analysis of variance. Linear regression relationships between N_mass_, N_area_, and LMA were obtained using individual leaves as replicates, and the effects of treatments on slopes and intercepts were evaluated using analysis of covariance. Where non-significant Ancova interaction indicated that slopes were not different, the interaction term was dropped from the model and intercepts were compared.

For analysis of the leaf shedding data, half of the 100% irradiance plants were assigned to the TRANS group to obtain a complete design, as described above. Analysis of covariance was performed, with mean day-of-year of leaf fall as the dependent variable, transfer treatment as main effect, and growth irradiance as continuous covariate. Because the interaction was not significant, it was excluded from the model and the analysis was repeated.

## Results

**Fig. 1:**
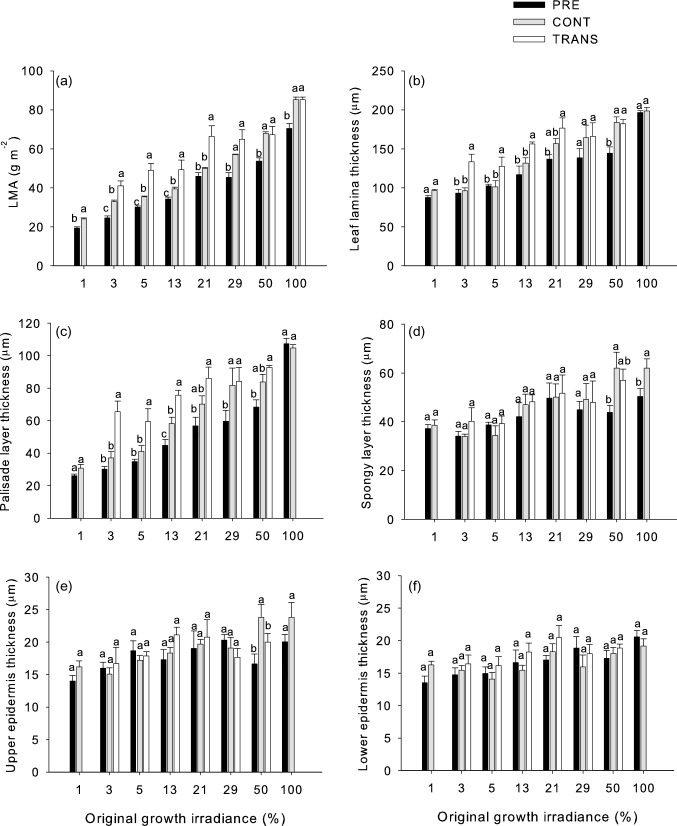
Responses of morpho-anatomical traits of mature leaves of *A. pseudoplatanus* seedlings to a sudden increase in irradiance. Means and standard errors for carbohydrate-free leaf mass per area (LMA (**a**)), leaf lamina thickness (**b**), palisade mesophyll thickness (**c**), spongy mesophyll thickness (**d**), thickness of upper (**e**) and lower (**f**) epidermis ae shown. Leaves were sampled on August 5, i.e., prior to transfer to full irradiance (PRE) and 30 days later (TRANS—transferred plants, CONT—plants left under the original irradiance). Significant contrasts between treatment groups within the same initial growth irradiance are indicated by different letters above bars. For analysis of variance see Table 1. Data for TRANS plants originally grown under lowest irradiance are missing because of the photo-oxidative damage to leaves

**Fig. 2:**
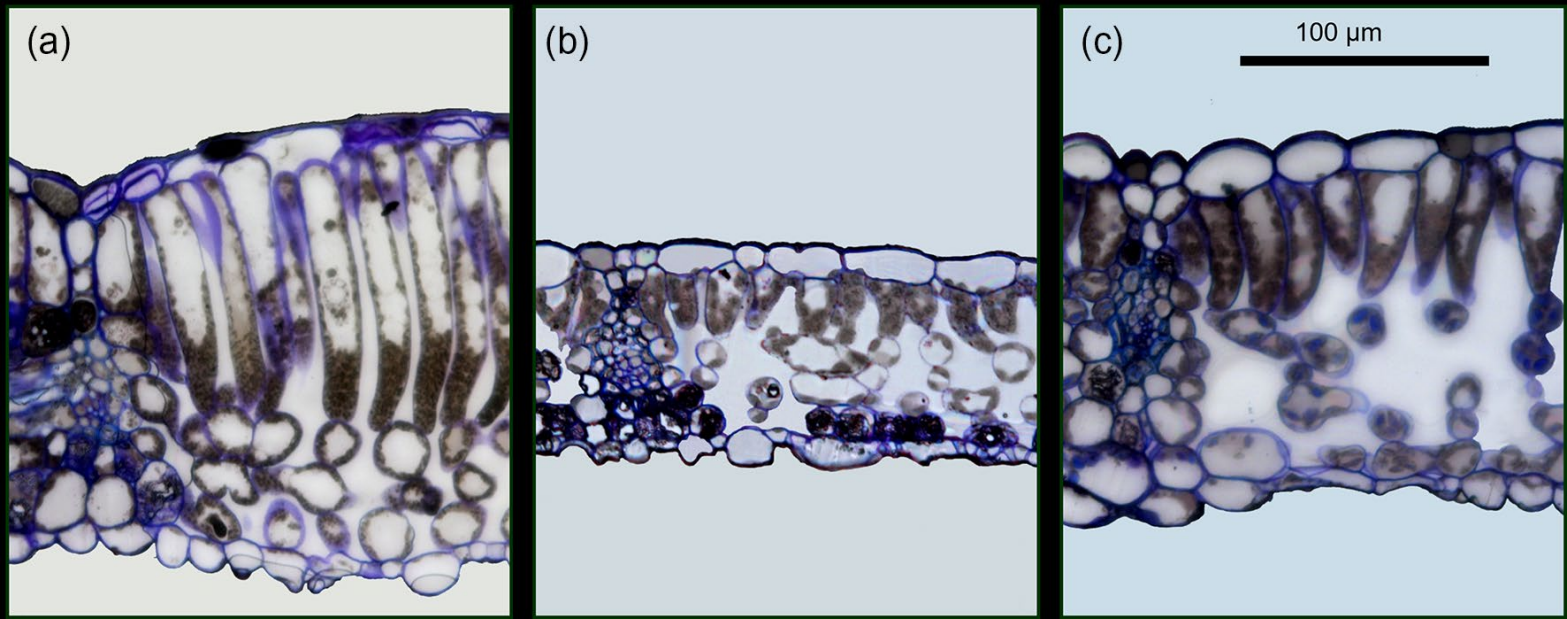
Anatomical adjustment of fully formed leaves *A. pseudoplatanus* in response to transfer from low to high irradiance. Light microscope images represent leaf cross-sections from plants maintained for the whole season at 100% irradiance (**a**), at 3% irradiance (**b**) and first grown at 3% and then transferred for 30 days to 100% irradiance (**c**). All samples were collected 30 days after transfer

**Fig. 3:**
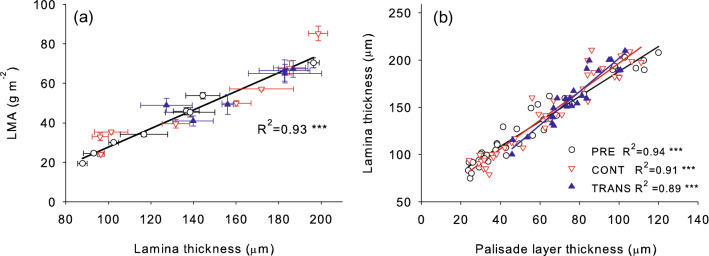
Linear relationships of leaf mass per area (LMA) vs. lamina thickness (**a**) and lamina thickness vs. palisade cell thickness (**b**) in *A. pseudoplatanus* leaves acclimated to eight levels of irradiance (PRE) and subsequently transferred to full solar irradiance (TRANS) or left under the original irradiance (CONT). In (**a**) points are means and bars indicate standard errors. In (**b**) values for individual leaves are plotted and lines are fitted to each treatment

**Fig. 4:**
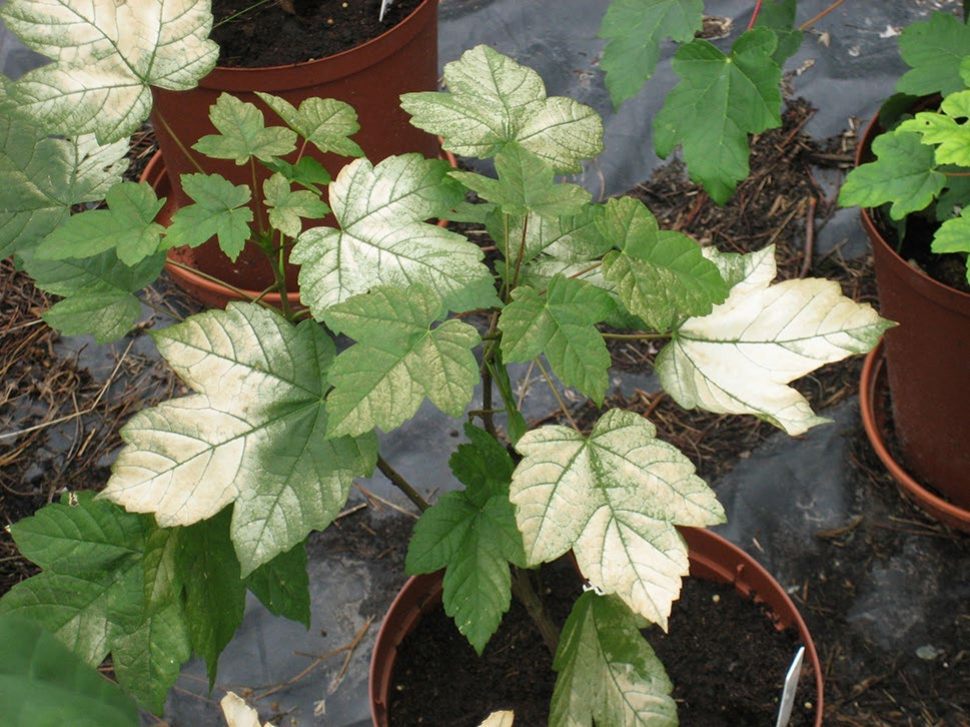
*A. pseudoplatanus* individual grown at 1% irradiance suffering photo-oxidative damage after transfer to 100% irradiance

Analysis of variance showed that LMA and all leaf tissues adjusted to growth irradiance (Table 1). LMA increased from lowest to highest PPFD without showing saturation (Fig. 1a). When plants maintained under the same shading intensities were resampled after 30 days (black vs. gray bars), the LMA value was significantly higher while retaining the positive relationship with irradiance. In plants that were transferred to full sun, LMA increased significantly more, except for those originating from the 29% and 50% treatment. Adjustment of LMA to growth irradiance and to change in irradiance was to a large extent caused by adjustment in lamina thickness (Figs. 1b, 2, 3a) that itself was strongly related to thickness of the palisade layer (Figs. 1c, 2, 3b). In particular, palisade cells became slightly longer in mature leaves between the two sampling dates at most irradiances and responded to transfer to full light with further elongation. This additional elongation response was especially strong in leaves formed at the lowest irradiance levels. Adjustment to growth irradiance was also found with respect to the thickness of spongy mesophyll, and upper and lower epidermis (Table 1; Fig. 1d–f). However, there was no clear increment between sampling dates, except for spongy layer and upper epidermis in the highest irradiances. There was also no clear effect of transfer to high light on the thickness of these three tissue layers. In plants transferred from 1% irradiance to full light, we observed rapid photobleaching (Fig. 4) and a subsequent leaf drop.

**Table 1:** Analysis of variance for the effects of original growth irradiance level (I, at 3, 5, 13, 21, 29, and 50% of solar irradiance, with 100% irradiance also included for LMA) and transfer treatment (T, at three levels: PRE, CONT, and TRANS) on morpho-anatomical traits

Trait	Source	DF	SS	F	*P*
LMA	I	6	10.535	110.152	** < 0.001**
	T	2	2.895	90.802	** < 0.001**
	I × T	12	0.408	2.133	**0.020**
Leaf thickness	I	5	2.524	27.612	** < 0.001**
	T	2	0.802	21.924	** < 0.001**
	I × T	10	0.228	1.248	0.280
Palisade thickness	I	5	5.085	33.903	** < 0.001**
	T	2	2.853	47.552	** < 0.001**
	I × T	10	0.433	1.444	0.183
Spongy layer thickness	I	5	1.637	6.407	** < 0.001**
	T	2	0.134	1.311	0.277
	I × T	10	0.382	0.749	0.676
Upper epidermis thickness	I	5	0.447	3.471	**0.008**
	T	2	0.046	0.890	0.416
	I × T	10	0.498	1.934	0.057
Lower epidermis thickness	I	5	0.468	4.030	**0.003**
	T	2	0.154	3.308	**0.043**
	I × T	10	0.111	0.477	0.899

Data were log transformed prior to analysis

Significant effects (*P* < 0.05) are highlighted by bold font

**Fig. 5:**
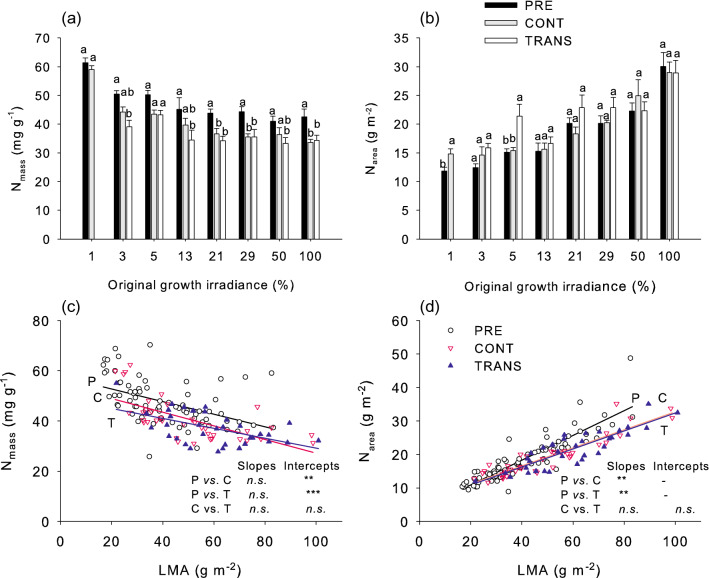
Effect of growth irradiance and sudden increase in irradiance on mean leaf N_mass_ (**a**) and N_area_ (**b**), and respective relationships between LMA and N_mass_ (**c**) and N_area_ (**d**) in mature *A. pseudoplatanus* leaves. Means ± standard errors are shown in (**a**) and (**b**) and individual leaf data in (**c**) and (**d**). For analysis of variance see Table 2; other details as in Fig. 1

Growth irradiance and transfer treatments significantly affected nitrogen contents, both when expressed on a leaf mass and on an area basis (Table 2). N_mass_ was inversely related to growth irradiance and declined across shading levels between sampling dates (Fig. 5a). In contrast, N_area_ was positively related to growth irradiance and showed no significant change between sampling dates (Fig. 5b). Transfer to full light did not significantly affect the N_mass_ but slightly and erratically increased N_area_ (Fig. 5a, b). The diversification of LMA and N contents by differences in growth irradiance in the first part of the growing season resulted in significant linear relationships between individual leaf LMA and N concentrations. These relationships were negative for mass-based, and positive for area-based concentrations (Fig. 5c, d). The slopes of N_mass_ vs. LMA relationships did not differ between treatments, however, the intercepts decreased between sampling dates (Fig. 5c). For the N_area_ versus LMA relationships, the slopes became reduced with time (Fig. 5d).

**Table 2:** Analysis of variance for the effects of original growth irradiance level (I, at 3, 5, 13, 21, 29, 50% and 100% of solar irradiance) and transfer treatment (T, at three levels: PRE, CONT, and TRANS) on leaf N concentration

Trait	Source	DF	SS	F	*P*
N (mg g^−1^)	I	6	0.845	7.511	** < 0.001**
	T	2	1.265	33.724	** < 0.001**
	I × T	12	0.079	0.353	0.977
N (g m^−2^)	I	6	6.157	29.463	** < 0.001**
	T	2	0.385	5.534	**0.005**
	I × T	12	0.456	1.092	0.374

Data were log transformed prior to analysis

Significant effects (*P* < 0.05) are highlighted by bold font

**Fig. 6:**
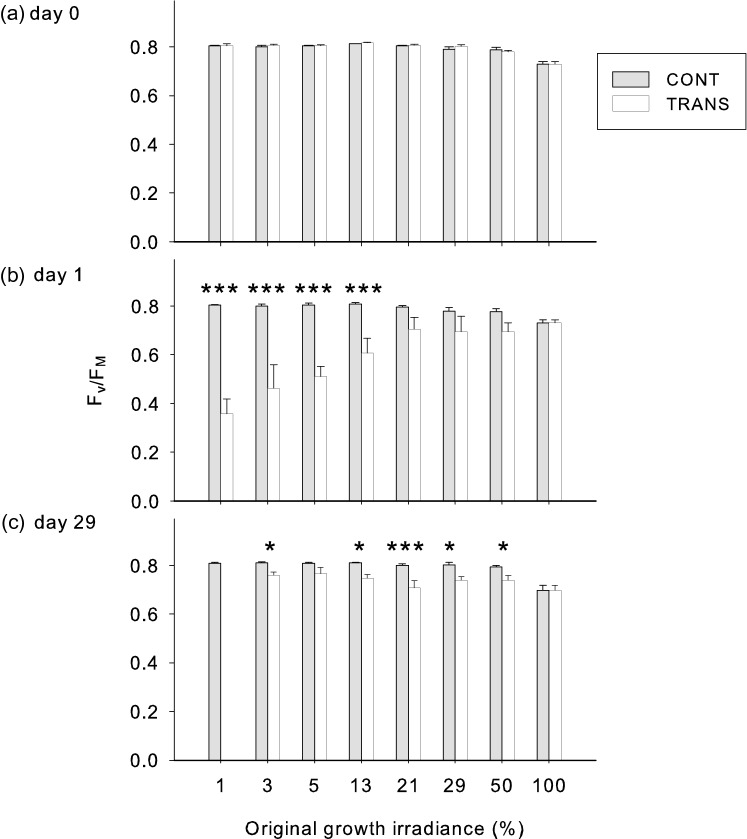
Efficiency of PSII in *A. pseudoplatanus* leaves acclimated to eight levels of irradiance measured immediately before transfer to full sun (**a**), one day after transfer (**b**) and 29 days after transfer (**c**). Means ± standard errors for plants transferred to full solar irradiance (TRANS) or remaining under the original irradiance (CONT) are shown. Asterisks indicate significant contrasts between treatments within each irradiance level *P < 0.05, ***P < 0.001

Immediately prior to transfer, the chlorophyll fluorescence parameter F_v_/F_M_ was above 0.80 in shaded plants up to 21% of full irradiance, with values in more strongly illuminated plants being somewhat lower (Fig. 6a). Transfer to full light resulted in overall reduction of F_v_/F_M_ (to as low as 0.35 in plants from 1% irradiance) and the magnitude of this decline was in reverse proportion to growth irradiance (Fig. 6b). However, by day 29 after transfer, the F_v_/F_M_ recovered to 0.70 or more, except for the 1% irradiance plants that had lost all leaves due to photobleaching (Figs. 4, 6c).

**Fig. 7:**
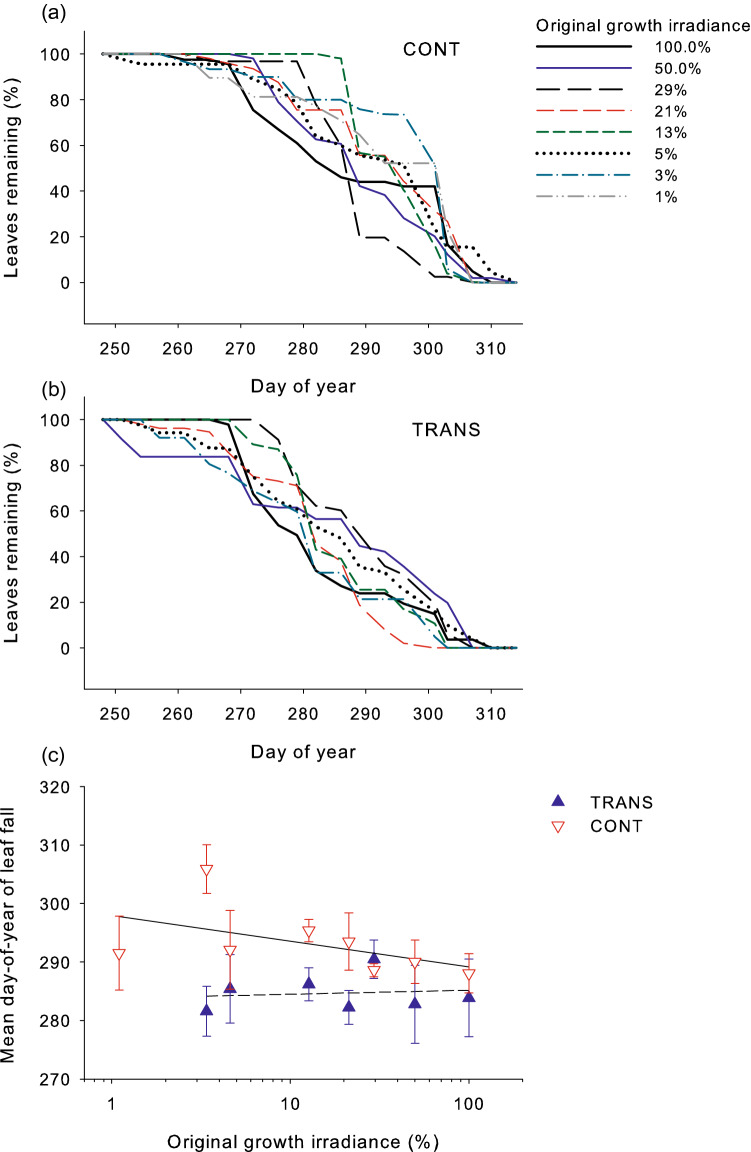
Effect of increased irradiance on autumnal shedding of leaves of *A. pseudoplatanus* plants acclimated to eight levels of irradiance. Proportion (%) of leaves remaining on plants in each treatment are plotted against day-of-year for plants remaining under the original growth irradiance (CONT (**a**)) and transferred to full solar irradiance (TRANS (**b**). In **c** the mean day-of-year of leaf fall is shown for each treatment in relations to initial growth irradiance. Lines in **a**, **b** show exact determinations for c. 50 leaves in each treatment. Means per plant ± standard errors (*N* = 5) are shown in (**c**). Slopes of the regression lines in (**c**) are not significantly different (Ancova interaction *P* = 0.259) and the common slope Ancova indicated lack of irradiance effect (*P* = 0.181) but there was a significant transfer effect (*P* = 0.004). Note that a log scale for x-axis was used in (**c**)

Autumnal leaf shedding began in early September, and was complete in early November. The duration of leaf retention on the plants in the autumn (measured as mean day-of-year of leaf fall) was scattered among irradiances (Fig. 7a, b), however, it showed no detectable relationship with irradiance intensity in TRANS (*R*^2^ = 0.33 *P* = 0.102) or CONT plants (*R*^2^ = 0.06 *P* = 0.820), or in both treatments analyzed jointly (Ancova growth irradiance effect *F*_1,13_ = 1.94 *P* = 0.181; Fig. 7c). The transfer to full light reduced leaf retention time across irradiances (Ancova transfer effect *F*_1,13_ = 12.57 *P* = 0.004; Fig. 7c).

## Discussion

In this study, we acclimated *A. pseudoplatanus* plants to eight levels of solar irradiance, from extreme shade to full sun. We then used these plants to test the ability of fully mature leaves developed under the different irradiance levels to re-adjust anatomically towards the high-light phenotype. The results were consistent with our leading hypothesis. Although leaves on plants grown in the deepest shade (1% irradiance) experienced severe photo-oxidative stress as indicated by bleaching, and died within a few days, anatomical re-acclimation was evident in plants from higher irradiances. It involved near-doubling of the palisade cell length in plants from the 3% irradiance and relatively smaller increases of palisade tissues in leaves from higher light levels. In contrast, neither spongy tissue nor any of the epidermal tissues increased significantly in thickness due to increased irradiance, making palisade tissue the sole structure capable of adjustment, and responsible for the increase in lamina thickness. It was likely also a major contributor to the increased LMA. This main result extends the list of known species capable of expanding palisade mesophyll in mature leaves (Bunce et al. 1977; Bauer and Thöni 1988; Yamashita et al. 2002; Amiard et al. 2005; Oguchi et al. 2005) and suggests that this mechanism may be more widespread than is accepted (Oguchi et al. 2018). Although our study species belongs to genus *Acer* in which a similar mechanism was found previously (Oguchi et al. 2005), it may be representative of an entire guild of late successional, shade-tolerant broadleaf trees, more of which should be investigated for similar capabilities.

The important question is whether and how this palisade cell growth contributes to dissipation of photo-oxidative stress and allows leaves to maintain or improve carbon uptake following a rapid increase in irradiance? The long palisade cells may facilitate acclimation of individual chloroplasts to local light intensity and provide additional volume for chloroplast placement and surface area for chloroplast contact with air spaces (Terashima et al. 2011). The high photosynthetic rates typical of sun leaves, however, are supported also by other anatomical features, such as densely spaced stomata and small veins, and a greater development of cell wall labyrinth in phloem parenchyma (Amiard et al. 2005; Poorter et al. 2019). Although we did not investigate most of these additional traits in our study, it is unlikely that new stomata or veins would have been formed upon exposure of mature leaves to stronger light. Thus, the additional thickness of palisade mesophyll would not have been matched by a larger number of stomata or by denser veins. It is interesting whether such structurally imbalanced leaves with sun-type palisade but retaining shade-type stomatal and vascular arrangement will nevertheless be able to perform faster photosynthesis. We posit that the answer to this question is yes, because increased photosynthetic capacity without accompanying anatomical changes has been documented in similarly designed studies (Mohammed and Parker 1999; Oguchi et al. 2006). However, it is also likely that such leaves will experience greater stomatal limitation of photosynthesis, especially under conditions of strong sunlight coupled with water stress. Leaf cooling ability may also be compromised. Additionally, given that the survival of sun leaves in microsites with high light, wind, and water deficit exposure is improved by the various mechanical structures, thicker cuticle, and more strongly developed epicuticular wax (Scoffoni et al. 2015; Coble and Cavaleri 2017), the acclimative potential of these traits in mature leaves needs to be examined.

Although direct measurements of photosynthetic rate were not performed, the recovery of variable fluorescence to a level only slightly lower than control following a transient but drastic decline, indicated that partial photosynthetic recovery took place concomitantly with palisade development. This recovery might have occurred through structural mechanisms, with, e.g., the expansion of palisade cells providing an increased contact area between chloroplasts and intercellular spaces to facilitate CO_2_ access (Oguchi et al. 2005, 2006; Terashima et al. 2006) but also through activation of photoprotection mechanisms, changes in pigment composition, and adjustments of other components of light harvesting and the carbon reduction cycle. The decline and recovery of Fv/F_M_ upon rapid increase in irradiance has been reported in juveniles of other tree species (Yamashita et al. 2000; Oguchi et al. 2006), but they were not necessarily accompanied by recovery of CO_2_ uptake (Yamashita et al. 2000), implying activation of other photo-protective mechanisms.

The role of palisade expansion versus biochemical adjustments in photosynthetic acclimation might be evaluated by studying the temporal dynamics of physiological versus anatomical acclimation, however, currently few data sets with sufficient temporal resolution exist. Increases in photosynthetic capacity in tree seedlings may be detected as soon as 5 days after increased exposure (Oguchi et al., 2006), whereas in an experiment with walnut an accumulation of N and enhancement of carboxylation capacity began within 4 weeks and was coordinated with a gradual increase in LMA (Frak et al. 2001). In our study, no significant accumulation of N was observed, however, leaf N appeared to be conserved in spite of the high-light stress and could possibly be reallocated during the photosynthetic re-acclimation process. Ultimately, the problem may be solved if tools permitting experimental manipulation of mesophyll development independently of external light level become available (Munekage et al. 2015).

Plasticity of the traits controlling light harvesting, such as crown structure, leaf arrangement, leaf size and shape, as well as the anatomical structure of leaves, is a salient element of the ecological strategy of late successional trees that typically regenerate in shady forest understories and reach canopy or subcanopy layers by utilizing variously sized canopy gaps (Ashton and Berlyn 1992; Oguchi et al. 2006). A mid- or late-season gap opening creates a problem of excessive irradiance as well as a chance to utilize the additional light in photosynthesis. The ability to expand the palisade cells in already-formed shade leaves may constitute a mechanism to cope with both. However, the time available for re-acclimation of leaf structure and function following canopy-gap formation during the growing season is limited, therefore, given the requirement for additional biomass allocation to the leaf structure, the benefit of such change is conditional. The fact that *A. pseudoplatanus* leaves from the transferred plants were shed earlier than control leaves suggests that the degree and rate of re-acclimation were not fully effective for protection against damage from photo-oxidative stress. Indeed, the re-acclimation at 30 days after transfer was still incomplete (e.g., palisade cells from transferred plants were still shorter than in leaves experiencing full irradiance during the whole season). Since our objective was to test leaves that were fully developed, i.e., not only expanded but also having fully formed cell walls, our experiment was conducted in the second half of the growing season. In similar experiments with walnuts, the timing of increased exposure was critical, with exposure at 58 d after bud burst resulting in significant structural and photosynthetic acclimation of existing leaves and exposure at 91 days resulting in no acclimation (Frak et al. 2001). It is thus likely that anatomical responses would have been stronger if the transfer to high light occurred significantly earlier, when cell walls were more elastic and there was more time remaining until autumn (Frak et al. 2001).

Although *A. pseudoplatanus* forms terminal buds relatively early in the growing season, it does have the ability for sylleptic (Lammas) growth, similarly to many other woody species. An alternative option to leaf re-acclimation is therefore the production of new sun-type leaves (however, often with a carry-over effect; Yamashita et al. 2000; Martinez and Friedley 2018). The making of new leaves is certainly more costly in terms of biomass expense than the adjustment of pre-existing leaves. The balance between costs and benefits should depend on the extent of photo-oxidative damage to pre-existing leaves (itself dependent on initial growth irradiance) and on the timing of gap formation with respect to the anticipated length of the growing season, resulting in varied expected photosynthetic income. A trade-off may be expected, and needs to be tested, between the ability to modify pre-existing leaves and the efficiency of production of new, additional leaves in response to increased irradiance.

Although the scenario we tested was equivalent to a sudden gap formation, light availability may also change slightly and gradually throughout the growing season due to upper canopy defoliation (Rozendal and Kobe 2016) and to the changing leaf or solar angle. Such changes may not induce sudden stress and allow a more gradual anatomical and physiological acclimation. Notably, our data indeed showed that some palisade cell expansion occurred also between August 5 and September 5 even in CONT plants. This effect was unexpected but was consistent with the higher number of daily sunshine hours during that period (see Supplementary Information Fig. S1). Adjustments of palisade cell length in developed leaves after increases in irradiance of various rate and magnitude may thus supplement the numerous mechanisms allowing acclimation to increased light in late successional species (Frak et al. 2001; Youngblood and Fergusson 2003; Oguchi et al. 2006; Wen et al. 2008; Cano et al. 2011).

By employing multiple levels of irradiance in our experimental design, our study provides additional insights into the relationships between light and variation in leaf anatomy. Such variation has been traditionally described in terms of sun/shade dichotomy (Givnish 1988). Only recently, information on dose–response relationships between irradiance and leaf traits has been summarized through a multi-species and multi-experiment meta-analysis (Poorter et al. 2019). These authors demonstrated that the relative increase of leaf mass per area, leaf thickness, leaf density, and stomatal density is strongest in low irradiance with an apparent saturation at higher irradiance levels. Their data set, however, combined plastic responses of individual species with the adaptive interspecific differences. Only a limited number of studies have attempted to determine the shape of analogous dose–response relationship for leaf anatomy within a single species. Most of such information has been provided by reports of leaf variability within the crowns of individual trees or along natural understory light gradients. Leaf mass per area as well as lamina thickness or, rarely, palisade thickness have been shown to follow a saturating mode of response to irradiance (Bond et al. 1999; Poorter 1999; Posada et al. 2009; Coble et al. 2014; Legner et al. 2014; Coble and Cavaleri 2017). Only rarely have linear (Niinemets 1997) or power (Sprugel et al. 1996) relationships been reported. Variation of leaf phenotype within the canopy may, however, be controlled by other environmental parameters covarying with irradiance, such as temperature or level of water stress, particularly important in the exposed canopy positions (Zwieniecki et al. 2004). Furthermore, the determination of irradiance at the leaf position in the canopy or under the canopy is frequently only approximate. The second type of studies uses controlled exposure of whole individuals, usually seedlings, to light, generally applying two or three irradiance levels (e.g., Strauss-Debenedetti and Berlyn 1994). Seldom have larger number of levels been used (five in each Sims et al. 1998 and Pons and Poorter 2014, six in Poorter 1999, eight in Babaei Soustani et al. 2014). Moreover, studies using multiple levels of irradiance tend to focus on integrated traits, such as leaf mass per area (or its reciprocal, specific leaf area) and lamina density and thickness, rather than detailed tissue structure. With our eight levels of irradiance, we found that the thickness of leaf lamina and palisade tissue showed no evidence of saturation within the ecologically relevant range of irradiance, similarly to the LMA. This finding is in contrast with the majority of the previously cited reports, mostly, however, based on within-canopy variation. Certainly, irradiance might not have been the only player in our experiment, as it probably co-varied with the frequency of occurrence of high temperature, vapor pressure deficit, and higher wind speed. All these factors alone, or by aggravating leaf-level water stress, are known to affect the leaf structure by increasing the leaf thickness (Wu et al. 2016; Oguchi et al. 2018) and the various components of density, especially the sizes of structural and vascular tissues (Poorter et al. 2009; Coble and Cavaleri 2015). Such multiple factors differ also among actual understory gaps, likely reinforcing the effect of increased irradiance. In our experiment, however, variability in leaf-level water stress was certainly reduced in comparison to canopies of mature trees or natural gaps due to regular watering and small plant size.

In summary, our study has demonstrated the ability of mature, shade-acclimated leaves of *Acer pseudoplatanus* to respond to increased irradiance by enlarging palisade cells resulting in increased leaf thickness. As part of the species’ ecological strategy, this mechanism has the potential to contribute to dissipation of photo-oxidative stress and to enable increased photosynthetic gain under greater availability of light. Both aspects need further study. The ability to re-acclimate mature leaves may be a significant component of the species’ response to gap formation during the growing season, with a potential bearing on forest dynamics and successional processes. In addition, we have documented a non-saturated light response relationship between growth irradiance and thickness of leaf lamina and palisade mesophyll, contrasting with the findings of the few analogous reports available.

## Supplementary Information

Below is the link to the electronic supplementary material.

Supplementary file1 (DOCX 105 kb)

## Data Availability

Data are available from https://doi.org/10.6084/m9.figshare.20348349
