# Supplementary material for: Anatomical acclimation of mature leaves to increased irradiance in sycamore maple (Acer pseudoplatanus L.)
Source: Photosynth Res. 2022 Sep 3;154(1):41–55. doi: 10.1007/s11120-022-00953-4 (PMC9568483; doi:10.1007/s11120-022-00953-4)
Supplement: Supplementary file 1 — Supplementary file1 (DOCX 105 kb) [file 11120_2022_953_MOESM1_ESM.docx]

**Supplementary Information for**

**Anatomical acclimation of mature leaves to increased irradiance in sycamore maple (*Acer pseudoplatanus* L.)**

By Tomasz P. Wyka*, Piotr Robakowski, Roma Żytkowiak, Jacek Oleksyn

- Corresponding author, Adam Mickiewicz University, Faculty of Biology, Institute of Experimental Biology, General Botany Laboratory, ul. Uniwersytetu Poznańskiego 6, 61-614 Poznań, Poland; twyka@amu.edu.,pl

**Photosynthesis Research**

**Table S1** Measurements of instantaneous photosynthetic photon flux density inside and outside of shade houses and relative irradiances estimated for each shade house (each N=5).

| Shade house number | PPFD inside  (µmol m^-2^ s^-1^) | | | PPFD outside  (µmol m^-2^ s^-1^) | | | Relative irradiance (%) | |
| --- | --- | --- | --- | --- | --- | --- | --- | --- |
|  | mean | S.D. | | mean | S.D. | | mean | S.D. |
| 1 | 829.20 | | 91.98 | 1686.00 | | 93.12 | 49.6 | 7.95 |
| 2 | 534.00 | | 54.53 | 1816.60 | | 102.31 | 29.4 | 3.19 |
| 3 | 396.80 | | 55.75 | 1840.60 | | 111.41 | 21.5 | 2.06 |
| 4 | 206.60 | | 41.24 | 1614.80 | | 91.54 | 12.7 | 1.93 |
| 5 | 73.40 | | 8.73 | 1599.20 | | 87.78 | 4.6 | 0.44 |
| 6 | 41.40 | | 1.95 | 1212.80 | | 15.17 | 3.4 | 0.14 |
| 7 | 14.00 | | 1.87 | 1217.20 | | 15.16 | 1.1 | 0.2 |

**Table S2** Heights (means, standard deviations, maximal and minimal values) of *Acer pseudoplatanus* plants at the start of the experiment (measured on May 4, 2009).

| Original growth irradiance (%) | N | Mean (cm) | S.D. | Minimum | Maximum |
| --- | --- | --- | --- | --- | --- |
| 100 | 10 | 37.4 | 6.9 | 27 | 47 |
| 50 | 10 | 39.9 | 15.8 | 21 | 74 |
| 29 | 10 | 40.5 | 12.4 | 24.5 | 58 |
| 21 | 10 | 29.3 | 10.6 | 17 | 44.5 |
| 13 | 10 | 41.1 | 15.9 | 12.5 | 65 |
| 5 | 10 | 33.4 | 12.3 | 15 | 60 |
| 3 | 10 | 38.7 | 14.5 | 17 | 67 |
| 1 | 10 | 42.0 | 11.4 | 27 | 63.5 |
| All irradiances | 80 | 37.6 | 12.9 | 12.5 | 74 |

| Growth irradiance  (% PPFD) | Treatment | LMA |  | Anatomical  traits |  | N_mass_ | N_area_ |  | F_v_/F_m_ D_0 | F_v_/F_m_ D_1 | F_v_/F_m_ D_29 |  | Leaf shedding |
| --- | --- | --- | --- | --- | --- | --- | --- | --- | --- | --- | --- | --- | --- |
| 100 | PRE | 10 |  | 7 |  | 10 | 10 |  |  |  |  |  |  |
| 100 | TRANS | 5 |  | 0 |  | 5 | 5 |  | 5 | 5 | 5 |  | 5 |
| 100 | CONT | 5 |  | 5 |  | 5 | 5 |  | 5 | 5 | 4 |  | 5 |
| 50 | PRE | 9 |  | 5 |  | 9 | 9 |  |  |  |  |  |  |
| 50 | TRANS | 5 |  | 4 |  | 5 | 5 |  | 4 | 5 | 5 |  | 5 |
| 50 | CONT | 5 |  | 5 |  | 5 | 5 |  | 5 | 5 | 5 |  | 5 |
| 29 | PRE | 9 |  | 4 |  | 9 | 9 |  |  |  |  |  |  |
| 29 | TRANS | 5 |  | 4 |  | 5 | 5 |  | 5 | 5 | 5 |  | 5 |
| 29 | CONT | 4 |  | 3 |  | 4 | 4 |  | 4 | 4 | 4 |  | 4 |
| 21 | PRE | 10 |  | 4 |  | 10 | 10 |  |  |  |  |  |  |
| 21 | TRANS | 5 |  | 3 |  | 5 | 5 |  | 4 | 5 | 5 |  | 5 |
| 21 | CONT | 5 |  | 5 |  | 5 | 5 |  | 5 | 5 | 5 |  | 5 |
| 13 | PRE | 9 |  | 4 |  | 9 | 9 |  |  |  |  |  |  |
| 13 | TRANS | 4 |  | 5 |  | 4 | 4 |  | 5 | 5 | 4 |  | 5 |
| 13 | CONT | 5 |  | 5 |  | 5 | 5 |  | 3 | 4 | 5 |  | 5 |
| 5 | PRE | 10 |  | 5 |  | 10 | 10 |  |  |  |  |  |  |
| 5 | TRANS | 5 |  | 4 |  | 5 | 5 |  | 5 | 4 | 3 |  | 5 |
| 5 | CONT | 5 |  | 5 |  | 5 | 5 |  | 5 | 5 | 5 |  | 5 |
| 3 | PRE | 9 |  | 5 |  | 9 | 9 |  |  |  |  |  |  |
| 3 | TRANS | 4 |  | 3 |  | 4 | 4 |  | 5 | 4 | 4 |  | 5 |
| 3 | CONT | 5 |  | 5 |  | 5 | 5 |  | 5 | 5 | 5 |  | 5 |
| 1 | PRE | 9 |  | 6 |  | 9 | 9 |  |  |  |  |  |  |
| 1 | TRANS | 0 |  | 0 |  | 0 | 0 |  | 3 | 5 | 0 |  | 0 |
| 1 | CONT | 5 |  | 5 |  | 5 | 5 |  | 5 | 5 | 5 |  | 5 |

**Table S3** Numbers of independent samples used for means calculation for particular variables in each growth irradiance and treatment. Note that in the 1% irradiance the transfered plants lost all leaves and were not sampled for most traits.

**Fig. S1** Weather data for the experimental site: daily hours of direct sunshine (a) and daily minimal (T_min_) and maximal (T_max_) temperatures (b). Dashed vertical lines indicate the leaf sampling dates (August 5 and Sept. 5).
